# Lysines and Arginines play non-redundant roles in mediating chemokine-glycosaminoglycan interactions

**DOI:** 10.1038/s41598-018-30697-y

**Published:** 2018-08-16

**Authors:** Prem Raj B. Joseph, Kirti V. Sawant, Junji Iwahara, Roberto P. Garofalo, Umesh R. Desai, Krishna Rajarathnam

**Affiliations:** 1 Department of Biochemistry and Molecular Biology, University of Texas Medical Branch, Galveston, TX 77555 USA; 2 Sealy Center for Structural Biology and Molecular Biophysics, University of Texas Medical Branch, Galveston, TX 77555 USA; 3 Department of Microbiology and Immunology, University of Texas Medical Branch, Galveston, TX 77555 USA; 4 Department of Pediatrics, University of Texas Medical Branch, Galveston, TX 77555 USA; 5 Department of Medicinal Chemistry and Institute for Structural Biology and Drug Discovery, Virginia Commonwealth University, Richmond, VA 23219 USA

## Abstract

Glycosaminoglycans (GAGs) bind a large array of proteins and mediate fundamental and diverse roles in human physiology. Ion pair interactions between protein lysines/arginines and GAG sulfates/carboxylates mediate binding. Neutrophil-activating chemokines (NAC) are GAG-binding proteins, and their sequences reveal high selectivity for lysines over arginines indicating they are functionally not equivalent. NAC binding to GAGs impacts gradient formation, receptor functions, and endothelial activation, which together regulate different components of neutrophil migration. We characterized the consequence of mutating lysine to arginine in NAC CXCL8, a well-characterized GAG-binding protein. We chose three lysines — two highly conserved lysines (K20 and K64) and a CXCL8-specific lysine (K67). Interestingly, the double K64R/K20R and K64R/K67R mutants are highly impaired in recruiting neutrophils in a mouse model. Further, both the mutants bind GAG heparin with higher affinity but show similar receptor activity. NMR and MD studies indicate that the structures are essentially identical to the WT, but the mutations alter the network of intramolecular ion pair interactions. These observations collectively indicate that the reduced *in vivo* recruitment is due to altered GAG interactions, higher GAG binding affinity can be detrimental, and specificity of lysines fine-tunes *in vivo* GAG interactions and function.

## Introduction

Glycosaminoglycans (GAGs) are polysaccharides that are ubiquitous components of cell surfaces and the extracellular matrix^1–4^. They bind hundreds of proteins including chemokines and growth factors, and mediate and regulate diverse fundamental roles in human pathophysiology from inflammation and organogenesis to cancer progression^5–9^. GAGs, such as heparan sulfate (HS) and heparin, are negatively charged linear sulfated polysaccharides of repeating 1–4 linked disaccharide units, made up of uronic acid and α-D-glucosamine^2^. It is well established that protein-HS/heparin interactions are primarily driven by ion pair interactions (salt bridges), between clusters enriched in lysines (Lys) and arginines (Arg) in the protein and sulfates/carboxylates in HS^2,10–13^. Whereas, arginines are enriched relative to lysines in protein-protein and protein-DNA interfaces^14,15^, sequences of some GAG-binding neutrophil-activating proteins reveal high selectivity for lysines over arginines^16^. Why lysines are preferred in these GAG-binding proteins and how this specificity impact structure, affinity, and function are not known.

**Figure 1:**
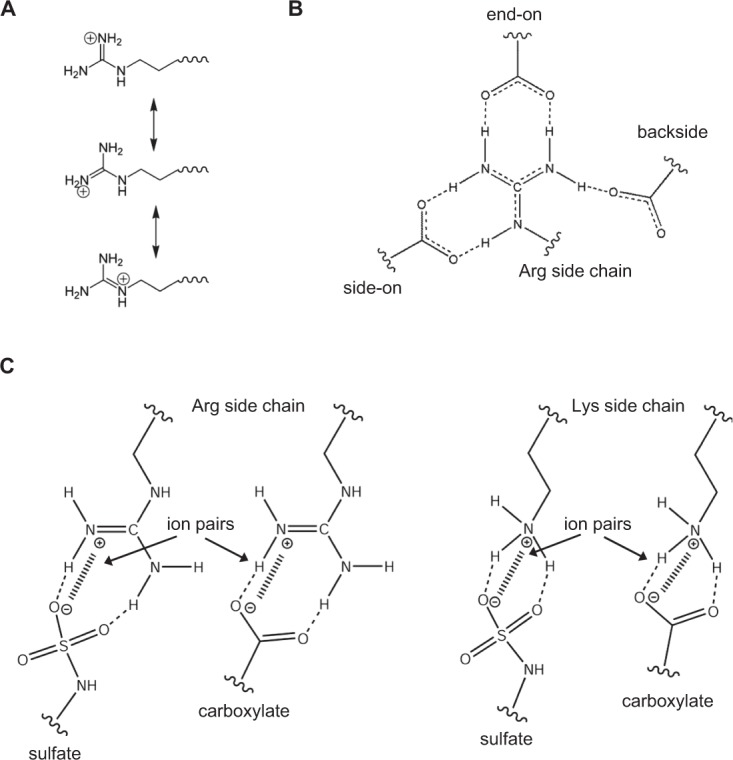
Interactions of lysines and arginines with sulfate/carboxylate groups. Schematics showing (**A**) Charge delocalization in the guanidinium group of arginine. (**B**) Different interactions possible for Arg side chain with carboxylates and (**C**) Lys and Arg ion pair interactions with sulfates and carboxylates.

The pKa of the surface lysine amino and arginine guanidinium groups are >10 indicating that they are always protonated and charged at physiological pH. In principle, lysines and arginines could be involved in short-range H-bonding (hydrogen-bonding) and ion pair interactions and long-range electrostatic interactions, and the energetics of these interactions can vary by many kilocalories^12,17–22^. The side chain structures, geometry, and proximity of the binding partners dictate binding energies. Whereas lysine amino NH_3_^+^ group is symmetric and has a simple structure, the guanidinium group of arginine is planar and asymmetric with a more complex structure (Fig. 1). The conjugation between the double bond and the nitrogen lone pairs in the guanidinium group results in delocalization of the positive charge allowing multiple ion pair and H-bonding interactions with sulfate/carboxylate groups that are less likely for the Lys NH_3_^+^ group (Fig. 1A)^12^. The arginine guanidinium group allows several potential interaction with carboxylates — (i) the side-on and end-on interactions (bidentate configuration) and (ii) the backside interaction (monodentate configuration) involving the Nη1 hydrogen closest to Nε (Fig. 1B)^17,23^. In addition, the guanidinium compared to the amino cation can form strong electrostatic interactions with sulfate anion based on Pearson’s concept of soft-acid soft-base interactions (Fig. 1C)^12,24^. In essence, the guanidinium group allows 5–6 H-bonds, while the spherically symmetric nature of the lysine amino group allows fewer and more dynamic H-bonds^12,17,25,26^.

Solvation plays a key role in dictating side chain interactions of arginines and lysines. Based on the classical Born’s model on solvation free energy and Collin’s model of charge density-dependent strength of hydration, it is energetically less costly to remove water from a large low-charge density gaunidinium ion (Arg) than from a small high-charge density amino ion (Lys)^27,28^. Therefore, desolvation energy is much smaller for arginine compared to lysine. In a recent MD study of a protein-DNA complex, it has been shown that the mean lifetimes of direct H-bond/ion pair interactions of arginine are much longer and less dynamic than those involving lysines^26^. Also, in the case of arginines, contact ion pairs can be more energetically favorable compared to lysines where both contact ion pairs and solvent separated ion pairs can alternate in a dynamic fashion.

**Figure 2:**
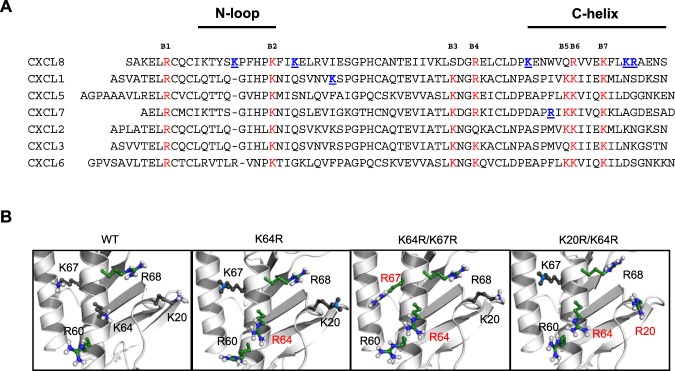
(**A**) Sequence alignment of neutrophil-activating chemokines. Conserved basic residues implicated in GAG interactions are highlighted in red. Chemokine-specific residues that are involved in heparin binding are shown in blue and underlined. (**B**) The location of the lysines and arginines in the WT structure and the arginine mutant structural models are shown. Substituted arginines are labelled in red.

A set of seven chemokines, characterized by the N-terminal ‘ELR’ motif (Fig. 2A), mediate neutrophil recruitment for combating microbial infection. A fundamental aspect of neutrophil-activating chemokine (NAC) function involves interactions with GAGs. GAG interactions affect gradient formation, receptor function, and endothelial activation^11,29–31^. Structural basis and identity of GAG-binding residues of four NACs, CXCL1, CXCL5, and CXCL7, and CXCL8, have been determined using solution NMR spectroscopy, molecular dynamics, mutagenesis, and functional studies^32–37^. Seven basic GAG-binding residues are conserved among the ELR-chemokines and are labeled B1 to B7 (Fig. 2A). Lysines at B2 and B7 are absolutely conserved in all seven; B6 is a lysine in all except CXCL8, and B3, B5, and B6 are conserved in at least five out of seven NACs. B4 shows no preference for lysines or arginines^16^. The conserved arginine (B1) is involved in GAG interactions only in CXCL1, but is a very critical residue for receptor activation for all seven chemokines, indicating that the major function of this conserved arginine is not for GAG binding. The preference for lysines stands out, suggesting that the structural features of lysine provide specificity that cannot be conferred by an arginine.

In this study, we ask why lysines are highly preferred at certain locations in NACs. We mutated three lysines to arginine in CXCL8 — two highly conserved lysines (K20 and K64) and a CXCL8-specific lysine (K67) (Fig. 2). The double mutants were significantly less active in trafficking neutrophils in a mouse model. The mutants bind GAG with higher affinity but show similar receptor activity, indicating that the reduced *in vivo* recruitment must be due to altered GAG interactions. NMR studies indicated that the tertiary structural fold of the single (K64R) and double (K64R/K20R and K64R/K67R) mutants is unaltered compared to the wide type (WT). Molecular dynamics (MD) studies show that the mutations alter the network of intramolecular interactions and result in increase in H-bond/ion pair donors of the GAG binding surface for the double mutants, but showed minimal change for the single mutant. These observations collectively indicate that the specificity imparted by a lysine cannot be replaced by an arginine, and that enhanced GAG binding as a result of arginine substitution is actually detrimental and impacts one or more of GAG-related functions that in turn impairs neutrophil recruitment function.

## Results

### Design and Characterization of Arginine Mutants

In CXCL8, the core GAG-binding domain has three lysines (K20, K64 and K67) and two arginines (R60 and R68)^32^. Among the lysines, K20 (B2) and K64 (B7) are absolutely conserved. K67 is not conserved, as other NACs besides CXCL2 have polar or acidic residues (Fig. 2A)^16^. Our NMR-based model of CXCL8-heparin octasaccharide shows that K64 lies in the middle of the binding surface suggesting it functions as an important anchor point, and mutating this lysine to alanine also significantly impairs neutrophil recruitment^32^. Therefore, we designed three mutants centered on K64 to address how conserved and chemokine-specific lysines influence structure and function — K64R mutant, a double K20R/K64R mutant that addresses the role of highly conserved lysines and a double K64R/K67R mutant that addresses the role of a highly conserved and CXCL8-specific lysine. The substitutions are shown in the models generated from the WT CXCL8 structure (PDB ID: 1IL8) (Fig. 2B).

**Figure 3:**
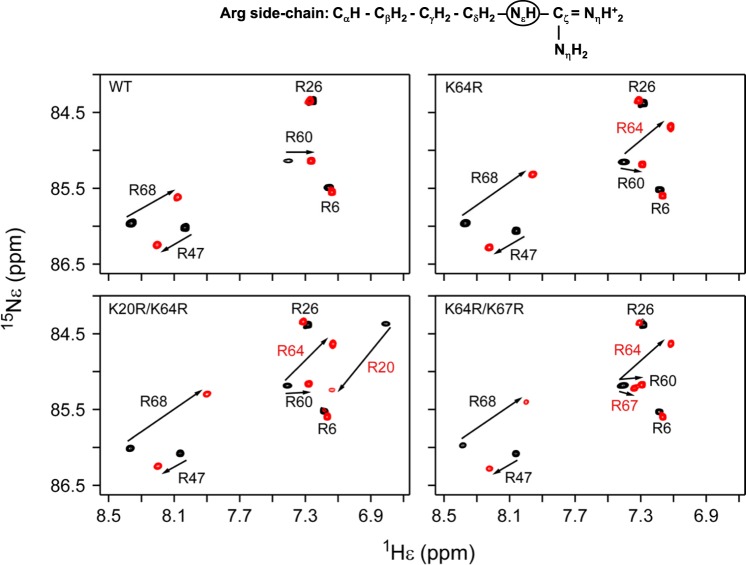
NMR characterization of arginine side chain interactions of the CXCL8 mutants with heparin dp8. Overlay of the Nε-Hε selective ^1^H-^15^N HISQC spectra of the arginine side chains in the free (black) and heparin dp8-bound complexes (red). Arrows connect the respective free and bound peak. A schematic of arginine side chain is shown on the top and the side chain NεH resonance detected in the HISQC spectra is circled.

The structural consequence of the substitutions was assessed from the ^1^H-^15^N heteronuclear single-quantum coherence (HSQC) spectra. The spectra of all the mutants were essentially identical to the WT spectrum, indicating that the arginines, despite the larger guanidinium group compared to the lysine amino group, are accommodated and do not perturb the tertiary fold except for minor local perturbations around the site of substitution (Supplementary Figs S1–S3). The arginine side chain spectra of the WT and mutants are shown in Fig. 3. Backbone amide and side chain Nε-Hε resonances of the newly introduced arginines (R20, R64, and R67) in the single and double mutants were assigned by comparing the ^1^H-^15^N HSQC and Nε-Hε selective ^1^H-^15^N HISQC spectra of the single and double mutants to the WT (Fig. 3 and Supplementary Figs S1–S3).

### Heparin binding of arginine mutants

We initially characterized GAG heparin octasaccharide (dp8) binding-induced chemical shift perturbations (CSPs) of arginine side chain Nε-Hε resonances. In the WT, the core GAG binding arginines R60, R68 and R47 show large perturbation. Residues R6 of the N-terminus and R26 on the first β-strand, which are on the opposite face of the GAG binding interface, are not perturbed or show minimal perturbation (Fig. 3). In the K64R, K64R/K67R, and K20R/K64R mutants, the newly introduced arginines show perturbation, indicating the substituted arginines (R20, R64 and R67) are involved in direct GAG interactions.

**Figure 4:**
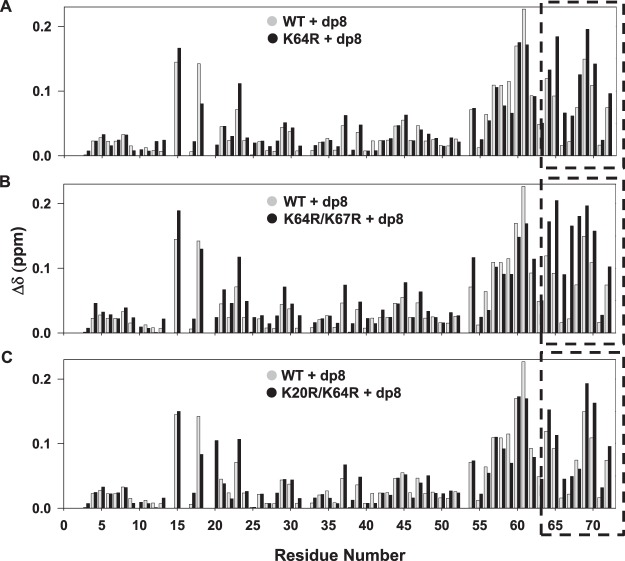
Backbone CSP histogram plots of the arginine mutants. Comparison of heparin dp8 binding-induced CSP of (**A**) K64R (**B**) K64R/K67R and (**C**) K20R/K64R mutants. The data for the mutants are shown in black and for the WT in gray. The dashed box indicates the C-terminal residues 64 to 72 showing relatively larger perturbation compared to WT.

With the knowledge that all the substituted Arg residues are involved in direct binding, we then compared the backbone perturbations of the Arg mutants. The backbone CSP provides insights into direct binding and indirect interactions including binding-induced structural changes, and so can provide a global picture of how specific mutations influence GAG binding of all other residues. The K64R and K64R/K67R mutants compared to the WT showed pronounced CSPs for the C-terminal helical residues R64 to S72 (Fig. 4A,B). The N-loop residues K15, H18 and K23 also showed CSP differences, while K20 did not show peak broadening as observed in the case of WT. The K20R/K64R mutant also showed increased perturbation, but less pronounced than in the case of K64R/K67R, for the C-terminal helical residues R64 to S72 except R68 (Fig. 4). Interestingly, R20 showed increased perturbation compared to K20 in the other mutants suggesting stronger H-bond/ion pair interactions. Overall, the backbone perturbations indicate altered network of interactions due to the arginine substitution in the C-helix and N-loop.

**Figure 5:**
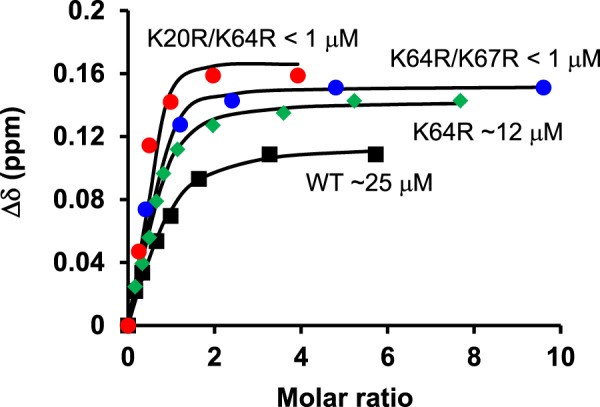
Heparin-binding affinities of the arginine mutants. Dissociation constants (K_d_) for the mutants were obtained by fitting the binding-induced chemical shift changes (Δδ) for a subset of well-resolved residues. A representative plot for residue E70 is shown along with the estimated dissociation constants (K_d_).

Binding affinities (K_d_) of WT and mutants to heparin dp8 were calculated from binding induced chemical shift changes of selected residues from the titration experiments. The difference in the affinities (K_d_) for the WT and the single mutant K64R was marginal (25 μM and 10 μM, respectively) (Fig. 5), while the K_d_ for the double mutants could not be accurately determined but is lower than 1 μM indicating arginine substitutions promotes tighter heparin interactions and higher binding affinity.

### Molecular Modeling and MD simulations of Arginine Mutants

Molecular Dynamics (MD) simulations can provide insights into atomic level details of how Lys to Arg substitutions impact spatial and dynamic features of the GAG binding surface. This exercise can provide an appreciation of changes in the surface characteristics due to altered intramolecular H-bonding/ion pair networks, surface electrostatics, concerted side chain motions, and potential changes in the availability of H-bond/ion pair donors due to the substituted arginines on the GAG binding surface. In particular, MD simulation of the mutants should provide useful insights into how arginine substitution results in altered C-terminal helical and N-loop interactions and tighter binding.

Structures of CXCL8 reveal five glutamate (Glu) residues in the proximity of the GAG binding Lys and Arg residues that could function as potential intramolecular ion pair/H-bonding partners. These include E29′ in the proximity of R68, K20 and K64 (′represents a residue in the second monomer across the dimer interface); E70 in the proximity of K67; E63 in the proximity of R60 and K64; E63′ in the proximity of K67 and K64; and E55 near K60. X-ray structure (PDB ID: 4XDX) shows two H-bonds between the side chains of R68 and E29′ (Nη2^…^Oε1 and Nε-H^…^Oε2) and a ion pair between K67 and E70 ((Nζ^…^Oε).

**Figure 6:**
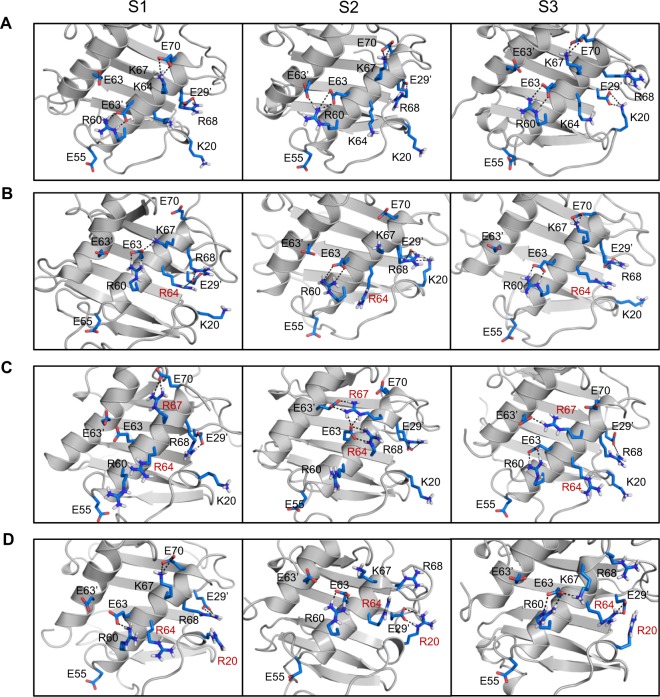
MD simulations of the CXCL8 arginine mutants. MD snapshots of the GAG-binding surface of (**A**) WT, (**B**) K64R, (**C**) K64R/K67R, and (**D**) K20R/K64R mutants. Intramolecular H-bonds/ion pairs between Lys/Arg and Glu residues over the course of the simulations are shown using dotted lines. S1, S2, and S3 are snapshots from three separate 5 ns trajectories extracted from each individual simulation. Each of the snapshots show distinctly different interaction pattern for the Arg and Lys residues. Substituted arginines are labelled in red.

Given that the structures of the mutants are essentially identical, models of the individual arginine mutants were generated from the WT dimer structure, energy minimized and then subjected to ~80–100 ns of *in silico* MD simulations using the Amber suite 14. Backbone rmsd vs. time profiles for the WT and mutants confirm that the analyzed trajectories represent stable relaxed structures (Fig. S4). In the case of WT CXCL8, R68 is involved in ion pair/H-bonding interactions with E29′, a feature similar to what is observed in the X-ray structure (Fig. 6A). K67 interacts with E70 in the helix (as in the X-ray structure) and also transiently with E63. K20 side chain forms ion pairs with E29′ side chain and H-bonds with S30′ backbone. R60 is involved in ion pair and H-bond interactions with E63 and transiently with E63′ and E55. K64 transiently interacts with E63 or E29′.

For the mutants, we first discuss the interactions involving the native Lys and Arg residues, followed by those involving the substituted Arg residues and how it alters the network of native interactions. In all the three mutants, R68 ion pair/H-bonding interactions are similar to those observed in the WT dimer (Fig. 6). R60 interactions in the K64R and K20R/K64R mutant are similar to the WT, whereas interaction of R60 with E63′ is missing in the K64R/K67R mutant (Fig. 6). K67 interactions in the K64R and K20R/K64R mutant are similar to the WT with minor alterations from the substitutions (Fig. 6B,D). K20 interactions with E29′ is transient in K64R and missing in the K64R/K67R mutant (Fig. 6B,C).

In the K64R and K20R/K64R mutant, R64 interacts with E29′ and also transiently with E63 (Fig. 6B,D). R20 in the K20R/K64R mutant is engaged in extensive ion pair/H-bonding interactions with E29′ (Fig. 6D). In essence, E29′ interacts with R64 and R68 and R20, resulting in a concerted interplay of interactions between the Arg residues and E29′. In the K64R/K67R mutant, R67 sterically restricts interactions of R64 with E29′ and E63′ (Fig. 6C). R67 in the K64R/K67R, when compared to the WT, is additionally engaged in interactions with E63 and E63′. Dynamic interplay of interactions is observed between R67 and the three Glu residues (E63, E63′, and E70) that form a triad (Fig. 6C). The loss of E63′ interactions with R60 and R64 are compensated by new interactions with R67.

A qualitative analysis of the role of solvent on H-bond/ion pair network of arginines and lysines on the GAG binding surface was also carried out (data not shown). In the case of arginine, contact ion pairs/H-bonds are stable and have longer lifetimes compared to lysines where both contact ion pairs and solvent separated ion pairs alternate dynamically. These observations are also consistent with a recent MD study on a protein-DNA complex^26^.

In summary, MD simulations show minimal rearrangement of GAG binding surface for the K64R mutant, while the double mutants show major alterations in the GAG binding surface due to an increase in the ion pair/H-bond network and an increase in the available H-bond/ion pair donors on the GAG binding surface. These changes, in essence, can result in reduced dynamics of the interacting surface and promote tighter binding of heparin oligosaccharides (Fig. 5).

### Activity of arginine mutants

**Figure 7:**
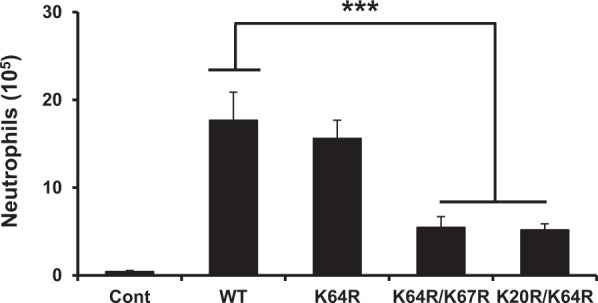
Neutrophil recruitment activity of the CXCL8 arginine mutants. The double mutants are significantly less active compared to the WT. The results are expressed as means ± S.E. from two independent experiments using 4 animals/group. Neutrophil levels for the mutants were compared to the WT using ANOVA followed by Tukey’s post hoc analysis. ***p < 0.001.

**Figure 8:**
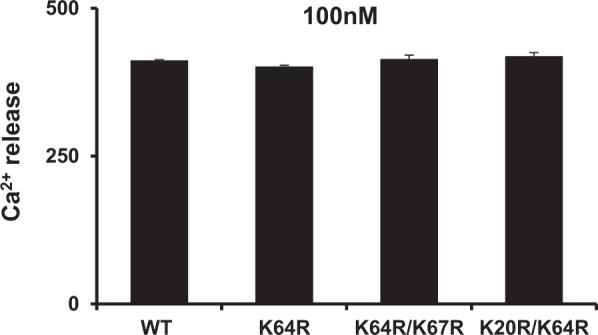
Calcium release activity of the CXCL8 arginine mutants. All the mutants had similar activity as the WT. Data shown are mean ± SEM of four measurements and is a representative of three independent experiments.

Neutrophil recruitment activity of the arginine mutants was measured in a peritoneal mouse model. Both K64R/K67R and K20R/K64R mutants showed highly impaired neutrophil recruitment activity compared to the wild type (WT) while the K64R mutant had comparable recruitment to the WT (Fig. 7). Considering the *in vivo* recruitment is the net result of both GAG and receptor interactions, we also characterized the *in vitro* CXCR2 activity of the Arg mutants. The calcium release activity of the arginine mutants was similar to the WT indicating that the mutations did not affect CXCR2 activation (Fig. 8). Therefore, altered *in vivo* recruitment of the Arg mutants must arise due to altered GAG interactions and not altered receptor activity.

## Discussion

Chemokine-mediated neutrophil recruitment from the blood to the infected/injury site must be highly coordinated for successful resolution^6–9^. Delayed recruitment or too few neutrophils could result in runaway inflammation and sustained recruitment or too many neutrophils could result in collateral tissue damage and disease^38,39^ Neutrophils must cross the endothelium and the extracellular matrix (ECM) to reach the insult site, and GAG interactions play a role in every single step of the way. GAGs are covalently linked to core proteins called proteoglycans (PGs). On the endothelium, PGs span the membrane and so GAGs are in the proximity of the membrane surface. In the ECM, PGs exist as macromolecular complexes with matrix proteins. Soluble and GAG-bound gradients direct neutrophils across the endothelium and the ECM to the insult site^11,29,40^. CXCL8 binding to endothelial GAGs results in activation of several signaling pathways, cytoskeletal changes, down regulation of gap junction proteins, and increase in permeability^41–43^. These changes promote and facilitate neutrophil migration across the endothelium. It has also been proposed that reorganization of the endothelial PGs facilitates neutrophil adhesion, an early critical step before crossing the endothelium^44,45^. Therefore, CXCL8-GAG interactions must be highly tuned for coordinated neutrophil recruitment to the insult site for successful resolution of infection.

Our current NMR study shows increased affinity of CXCL8 for heparin oligosaccharide as a consequence of arginine substitution. The question arises as to whether the mutations impact the monomer-dimer equilibrium either in the free or GAG-bound forms. The mutated residues are located away from the dimer interface and so it does not influence the dimerization constant of the free form. On the other hand, the higher GAG affinity of arginine mutants compared to WT does shift the GAG bound monomer-dimer equilibrium to the dimeric state. Any changes in function of the mutants can only be attributed to the GAG binding properties and not to some altered property of the mutants. This is clearly evident from the similar calcium release activity data for the WT and mutants (Fig. 8).

Several studies for CXCL8 and other NACs have shown alanine (Ala) mutants of lysines and arginines, which show weaker *in vitro* GAG binding, al**s**o show impaired *in vivo* neutrophil recruitment^8,46–51^. Interestingly, our current studies show that increased GAG affinity as a consequence of arginine substitution also impairs *in vivo* neutrophil recruitment indicating increased *in vivo* GAG affinity alters how GAG interactions regulate CXCL8-mediated neutrophil trafficking to the target tissue. Altered GAG interactions could impact duration and steepness of the gradients and/or endothelial activation that includes signaling and PG crosslinking^41,42,45^. Our future studies will focus on understanding how differences in lysine and arginine interactions impact these CXCL8-mediated GAG functions.

What could be the molecular basis underlying specificity? Considering lysines and arginines play fundamental roles in protein folding, protein-protein, and protein-nucleic acid interactions, how differences in lysine amino and arginine guanidinium groups influence ion pair interactions have been extensively studied and characterized from structural, computational, and mutational studies^12,17,26,52,53^. These studies collectively indicate differences in side chain structures could impact affinity, kinetics, and dynamics of binding interactions. Factors that influence these properties arise due to intrinsic differences in the H-bond/ion pair interactions and also as a function of their location in the macromolecular complex. NMR studies of protein-DNA complexes have shown that the dynamic characteristics of lysine and arginine side chains are quite different, and that the lysine amino groups continue to be dynamic whereas guanidinium group is less dynamic in the bound form, and that interactions involving the Arg guanidinium group are enthalpically favored and those involving lysine amino group are entropically favored^26^. Insights into the dynamic and kinetic characteristics of the individual lysines and arginines in protein-GAG complexes are lacking at this time, and such knowledge will lead to a better understanding on the selectivity of lysines and arginines and how they ultimately mediate GAG binding and function.

MD studies of the Arg mutants indicate that lysines and arginines in the GAG binding surface are involved in a network of intramolecular ion pair interactions, and therefore, the role of a basic residue must also be considered in concert with other residues in the binding surface. A more detailed and extensive characterization of mutations including those of arginines to lysines and similar studies in related chemokines and other GAG-binding proteins are necessary to fully appreciate the crosstalk between GAG-binding lysines and arginines in determining binding and function.

## Material and Methods

### Expression and Purification of CXCL8 Arginine mutants

Clones of the arginine mutants K64R, K20R/K64R and K64R/K67R were generated by PCR amplification using the QuikChange site-directed mutagenesis kit (Stratagene). Expression, and purification of CXCL8 WT and the mutants were carried out as described previously^54^. ^15^N-labeled proteins were prepared by growing cells in a minimal medium containing ^15^NH_4_Cl as the sole nitrogen source. Briefly, transformed cells were grown to an *A*_600_ ~ 0.6, and induced with 1 mM isopropyl β-D-thiogalactopyranoside (IPTG) overnight at 23 °C. The purity and molecular weight of the proteins were confirmed using matrix assisted laser desorption/ionization mass spectrometry.

### Molecular modeling and MD simulations

Models of the arginine mutants were generated by replacing the lysines with arginines using the mutagenesis module of Pymol 1.4^55^. The structures were subjected to multiple cycles of constrained and free energy minimizations using the AMBER 14 suite software to remove steric hindrances introduced by the mutations^56,57^. The energy-minimized structures were subjected to an equilibration protocol in explicit solvent^57^, and ~80 ns of MD production runs were carried out using the PMEMD (*Particle mesh Ewald molecular dynamics*) module of the AMBER 14 software suite on the Lonestar 5 CRAY XC40 cluster at the Texas Advanced Computing Center (TACC, UT Austin). Analysis of the trajectory was carried out using the Ambertools12 and the VMD molecular visualization software^56,58^. Molecular plots were prepared using Pymol^55^. The ion pairs were evaluated according to the distance between the donor (Nζ of Lys; Nε, Nη_1_ and Nη_2_ of Arg; Nδ_1_ and Nε_2_ of His) and acceptor atoms (Oε_1_ and Oε_2_ of Glu; Oδ_1_ and Oδ_2_ of Asp)^20^. If the distance (d) is <4.0 Å, the pair is counted as a ion pair. An ion pair is counted as a hydrogen bonding (H-bonding) pair when the geometry is acceptable (d < 3.5 Å, N-H^…^O angle > 120°).

### NMR Spectroscopy

For the ^1^H-^15^N HSQC titrations experiments, ^15^N-labeled proteins were prepared in 50 mM sodium phosphate buffer pH 7.0, 1 mM sodium azide, and 10% ^2^H_2_O (v/v). The starting protein concentrations were ~50 to 100 μM for the different experiments. Heparin octasaccharide (dp8) was purchased from Iduron (Manchester, UK). A 10 mM stock solution of dp8 was titrated into the protein sample in aliquots, and a series of ^1^H-^15^N HSQC spectra was collected at 30 °C until essentially no changes in chemical shifts were observed. The final protein:dp8 molar ratios for all titrations were ~1:10. The arginine side chain Nε-Hε resonances were recorded for the free and dp8 bound CXCL8 mutants using the Nε-Hε selective ^1^H-^15^N heteronuclear in-phase single quantum coherence (HISQC) pulse sequence^26^. By using ^15^N carrier position at 85 ppm together with selective pulses, the Arg side chain ^15^Nε-^1^Hε resonances were selectively observed in the HISQC spectrum. 100 μl of D_2_O was separately sealed for NMR lock in a coaxial insert (Norell Inc.). The free CXCL8 and dp8 bound samples were prepared in 20 mM sodium succinate, pH 5.4, and 0.4 mM NaF. The spectra were recorded at 40 °C. NMR experiments were acquired on a Bruker Avance III 800 MHz (equipped with a TXI cryoprobe) or 600 MHz (with QCI probe) spectrometer.

### Animals

Female, 7–8 week old, BALB/c mice (Harlan, Houston, TX) were maintained in specific-pathogen-free conditions in the animal research facility of UTMB, in accordance with NIH and UTMB institutional guidelines for animal care. Cages, bedding, food and water were sterilized before use. All animal work was approved by the Institutional Animal Care and Use Committee of the University of Texas Medical Branch at Galveston (Protocol: 0702005 C).

### Peritoneal neutrophil recruitment

Mice were inoculated intraperitoneally with 1 μg of arginine mutants in 100 μl of DPBS buffer using a 26 gauge needle. The mice were anesthetized 3 h post injection and euthanized by cervical dislocation. The peritoneal cavity was flushed twice with cold PBS, the harvested cells were centrifuged, and the pellet was suspended in PBS for cytospin slides. Slides were fixed, stained with Wright-Giemsa stain, and a differential leukocyte count was performed. Total neutrophils were counted using a hemocytometer after staining with Turk’s solution.

### Intracellular Ca^2+^ mobilization

Receptor activity of the mutants was measured from changes in intracellular Ca^2+^ levels using the Calcium 6 assay kit (FLIPR No-wash kit, Molecular Devices, Sunnyvale, CA) on a FlexStation III scanning fluorometer. In brief, CXCR2 transfected, differentiated HL60 (dHL60) cells were suspended in HBSS containing 10 mM HEPES and plated in flat-bottomed black microtiter plate (2 × 10^5^cells/well). The cells were loaded with FLIPR Calcium 6 dye following the manufacturer’s protocol for 1 h. 10 nM of CXCL8 arginine mutants prepared in HBSS buffer were transferred to the compound plates. Changes in fluorescence were monitored (λex 485 nm, λem 525 nm) every 5 s for 240 s at room temperature after automated addition of the mutants to the cells. The agonist response was determined by expressing the maximum change in fluorescence in arbitrary units over baseline.

## Electronic supplementary material

Supplementary figures

## References

[1] Li L, Ly M, Linhardt RJ (2012). Proteoglycan sequence. Mol. Biosyst.

[2] Xu D, Esko JD (2014). Demystifying heparan sulfate-protein interactions. Ann. Rev. Biochem..

[3] Schaefer, L. & Schaefer, R. M. Proteoglycans: from structural compounds to signaling molecules. *Cell Tissue Res*. **339** (2010). 10.1007/s00441-009-0821-y 19513755

[4] Gandhi NS, Mancera RL (2008). The structure of glycosaminoglycans and their interactions with proteins. Chem. Biol. Drug Des..

[5] Handel TM, Johnson Z, Crown SE, Lau EK, Proudfoot AE (2005). Regulation of protein function by glycosaminoglycans–as exemplified by chemokines. Ann. Rev. Biochem..

[6] Kang I, Chang MY, Wight TN, Frevert CW (2018). Proteoglycans as Immunomodulators of the Innate Immune Response to Lung Infection. J. Histochem. Cytochem..

[7] Hayashida K, Parks WC, Park PW (2009). Syndecan-1 shedding facilitates the resolution of neutrophilic inflammation by removing sequestered CXC chemokines. Blood.

[8] Frevert CW (2002). Tissue-specific mechanisms control the retention of IL-8 in lungs and skin. J. Immunol..

[9] Wang L, Fuster M, Sriramarao P, Esko JD (2005). Endothelial heparan sulfate deficiency impairs L-selectin- and chemokine-mediated neutrophil trafficking during inflammatory responses. Nature Immunol..

[10] Hileman RE, Fromm JR, Weiler JM, Linhardt RJ (1998). Glycosaminoglycan-protein interactions: definition of consensus sites in glycosaminoglycan binding proteins. Bioessays.

[11] Monneau Y, Arenzana-Seisdedos F, Lortat-Jacob H (2016). The sweet spot: how GAGs help chemokines guide migrating cells. J. Leukoc. Biol..

[12] Fromm JR, Hileman RE, Caldwell EE, Weiler JM, Linhardt RJ (1995). Differences in the interaction of heparin with arginine and lysine and the importance of these basic amino acids in the binding of heparin to acidic fibroblast growth factor. Arch. Biochem. Biophys..

[13] Fromm JR, Hileman RE, Caldwell EEO, Weiler JM, Linhardt RJ (1997). Pattern and spacing of basic amino acids in heparin binding sites. Arch. Biochem. Biophys..

[14] Tsai CJ, Lin SL, Wolfson HJ, Nussinov R (1997). Studies of protein-protein interfaces: a statistical analysis of the hydrophobic effect. Protein Sci..

[15] Nadassy K, Wodak SJ, Janin J (1999). Structural features of protein-nucleic acid recognition sites. Biochemistry.

[16] Rajarathnam K, Sepuru KM, Joseph PRB, Sawant KV, Brown AJ (2018). Glycosaminoglycan Interactions Fine-Tune Chemokine-Mediated Neutrophil Trafficking: Structural Insights and Molecular Mechanisms. J. Histochem. Cytochem..

[17] Musafia B, Buchner V, Arad D (1995). Complex salt bridges in proteins: statistical analysis of structure and function. J. Mol. Biol..

[18] Barlow DJ, Thornton JM (1983). Ion-pairs in proteins. J. Mol. Biol..

[19] Kumar S, Nussinov R (2002). Close-range electrostatic interactions in proteins. Chembiochem.

[20] Xu D, Tsai CJ, Nussinov R (1997). Hydrogen bonds and salt bridges across protein-protein interfaces. Protein Eng..

[21] Iwahara J, Esadze A, Zandarashvili L (2015). Physicochemical Properties of Ion Pairs of Biological Macromolecules. Biomolecules.

[22] Sankaranarayanan NV, Nagarajan B, Desai UR (2018). So you think computational approaches to understanding glycosaminoglycan-protein interactions are too dry and too rigid? Think again!. Curr. Opin. Struct. Biol..

[23] Donald JE, Kulp DW, DeGrado WF (2011). Salt bridges: geometrically specific, designable interactions. Proteins.

[24] Pearson RG (1963). Hard and Soft Acids and Bases. J. Amer. Chem. Soc..

[25] Luscombe NM, Laskowski RA, Thornton JM (2001). Amino acid-base interactions: a three-dimensional analysis of protein-DNA interactions at an atomic level. Nucleic Acids Res..

[26] Esadze A (2016). Changes in conformational dynamics of basic side chains upon protein-DNA association. Nucleic Acids Res..

[27] Honig B, Nicholls A (1995). Classical electrostatics in biology and chemistry. Science.

[28] Collins KD (1997). Charge density-dependent strength of hydration and biological structure. Biophys. J..

[29] Massena S (2010). A chemotactic gradient sequestered on endothelial heparan sulfate induces directional intraluminal crawling of neutrophils. Blood.

[30] Stoler-Barak L (2014). Blood vessels pattern heparan sulfate gradients between their apical and basolateral aspects. PloS one.

[31] Nourshargh S, Hordijk PL, Sixt M (2010). Breaching multiple barriers: leukocyte motility through venular walls and the interstitium. Nat. Rev. Mol. Cell. Biol..

[32] Joseph PR, Mosier PD, Desai UR, Rajarathnam K (2015). Solution NMR characterization of chemokine CXCL8/IL-8 monomer and dimer binding to glycosaminoglycans: structural plasticity mediates differential binding interactions. Biochem. J..

[33] Sepuru KM, Nagarajan B, Desai UR, Rajarathnam K (2016). Molecular Basis of Chemokine CXCL5-Glycosaminoglycan Interactions. J. Biol. Chem..

[34] Sepuru KM, Rajarathnam K (2016). CXCL1/MGSA Is a Novel Glycosaminoglycan (GAG)-binding Chemokine: Structural Evidence for Two Distinct Non-Overlapping Binding Domains. J. Biol. Chem..

[35] Brown, A. J., Sepuru, K. M. & Rajarathnam, K. Structural Basis of Native CXCL7 Monomer Binding to CXCR2 Receptor N-Domain and Glycosaminoglycan Heparin. *Int. J. Mol. Sci*. **18** (2017). 10.3390/ijms18030508 PMC5372524 28245630

[36] Brown AJ, Sepuru KM, Sawant KV, Rajarathnam K (2017). Platelet-Derived Chemokine CXCL7 Dimer Preferentially Exists in the Glycosaminoglycan-Bound Form: Implications for Neutrophil-Platelet Crosstalk. Front. Immunol..

[37] Joseph PR, Poluri KM, Sepuru KM, Rajarathnam K (2015). Characterizing protein-glycosaminoglycan interactions using solution NMR spectroscopy. Methods Mol. Biol..

[38] Bhatia M, Zemans RL, Jeyaseelan S (2012). Role of chemokines in the pathogenesis of acute lung injury. Amer. J. Resp. Cell Mol. Biol..

[39] Pinheiro da Silva F, Soriano FG (2009). Neutrophils recruitment during sepsis: Critical points and crossroads. Front. Biosci. (LandmarkEd).

[40] Rot A (1993). Neutrophil attractant/activation protein-1 (interleukin-8) induces *in vitro* neutrophil migration by haptotactic mechanism. Eur. J. Immunol..

[41] Derler, R. *et al*. Glycosaminoglycan-Mediated Downstream Signaling of CXCL8 Binding to Endothelial Cells. *Int. J. Mol. Sci*. **18** (2017). 10.3390/ijms18122605 PMC5751208 29207576

[42] Yan Z, Liu J, Xie L, Liu X, Zeng Y (2016). Role of heparan sulfate in mediating CXCL8-induced endothelial cell migration. PeerJ.

[43] Pichert A, Schlorke D, Franz S, Arnhold J (2012). Functional aspects of the interaction between interleukin-8 and sulfated glycosaminoglycans. Biomatter.

[44] Schmidt EP, Lee WL, Zemans RL, Yamashita C, Downey GP (2011). On, around, and through: neutrophil-endothelial interactions in innate immunity. Physiology (Bethesda).

[45] Dyer DP (2017). Differential structural remodelling of heparan sulfate by chemokines: the role of chemokine oligomerization. Open Biol..

[46] Kuschert GS (1998). Identification of a glycosaminoglycan binding surface on human interleukin-8. Biochemistry.

[47] Sawant KV (2016). Chemokine CXCL1 mediated neutrophil recruitment: Role of glycosaminoglycan interactions. Sci. Rep..

[48] Frevert CW (2003). Binding of interleukin-8 to heparan sulfate and chondroitin sulfate in lung tissue. Amer. J. Resp. Cell Mol. Biol..

[49] Tanino Y (2010). Kinetics of chemokine-glycosaminoglycan interactions control neutrophil migration into the airspaces of the lungs. J. Immunol..

[50] Rajasekaran D (2012). A model of GAG/MIP-2/CXCR2 interfaces and its functional effects. Biochemistry.

[51] Gangavarapu P (2012). The monomer-dimer equilibrium and glycosaminoglycan interactions of chemokine CXCL8 regulate tissue-specific neutrophil recruitment. J. Leukoc. Biol..

[52] Sokalingam S, Raghunathan G, Soundrarajan N, Lee SG (2012). A study on the effect of surface lysine to arginine mutagenesis on protein stability and structure using green fluorescent protein. PloS one.

[53] Sokalingam S, Madan B, Raghunathan G, Lee SG (2013). *In silico* study on the effect of surface lysines and arginines on the electrostatic interactions and protein stability. Biotechnol. Bioproc. E..

[54] Fernando H, Nagle GT, Rajarathnam K (2007). Thermodynamic characterization of interleukin-8 monomer binding to CXCR1 receptor N-terminal domain. FEBS J..

[55] DeLano, W. L. The Pymol Users Manual, DeLano Scientific LLC, Palo Alto, CA. (2002).

[56] Case, D. A. *et al*. AMBER 12, University of California, San Francisco. (2012).

[57] Lee MC, Deng J, Briggs JM, Duan Y (2005). Large-scale conformational dynamics of the HIV-1 integrase core domain and its catalytic loop mutants. Biophys. J..

[58] Humphrey W, Dalke A, Schulten K (1996). VMD: visual molecular dynamics. J. Mol. Graphics.

