# Supplementary material for: Lysines and Arginines play non-redundant roles in mediating chemokine-glycosaminoglycan interactions
Source: Sci Rep. 2018 Aug 16;8:12289. doi: 10.1038/s41598-018-30697-y (PMC6095893; doi:10.1038/s41598-018-30697-y)
Supplement: Supplementary file 1 — Supplementary figures [file 41598_2018_30697_MOESM1_ESM.pdf]

SUPPLEMENTARY MATERIAL

Lysines and Arginines play non-redundant roles in mediating chemokine-glycosaminoglycan interactions

Prem Raj B. Joseph[1,2], Kirti V. Sawant[1,2], Junji Iwahara[1,2], Roberto P. Garofalo[3,4], Umesh R. Desai[5], and Krishna Rajarathnam[1,2,3*]

[1]Department of Biochemistry and Molecular Biology, University of Texas Medical Branch, Galveston, Texas TX 77555, USA
[2]Sealy Center for Structural Biology and Molecular Biophysics, University of Texas Medical Branch, Galveston, TX 77555, USA.
[3]Department of Microbiology and Immunology, University of Texas Medical Branch, Galveston, TX 77555, USA.
[4]Department of Pediatrics, University of Texas Medical Branch, Galveston, TX 77555, USA.
[5] Department of Medicinal Chemistry and Institute for Structural Biology and Drug Discovery, Virginia Commonwealth University, Richmond, VA 23219, USA.

[*]Correspondence to: Krishna Rajarathnam, University of Texas Medical Branch, 301 University Blvd., Galveston TX 77555; Tel: 409-772-2238; Fax: 409-772-1790; E-mail: krrajara@utmb.edu

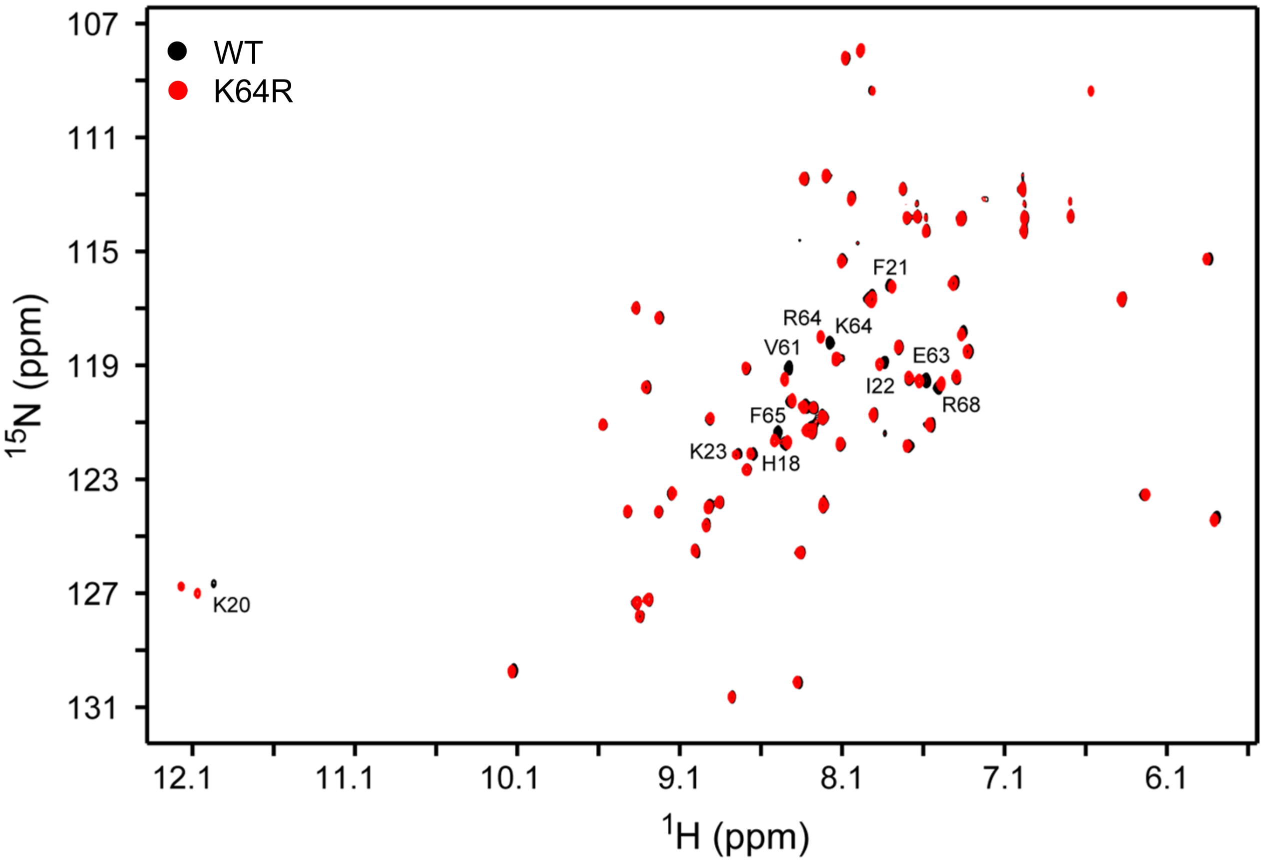

Figures S1. NMR structural characteristics of the K64R mutant. ^{1}H-^{15}N HSQC spectrum of K64R (red) overlaid on WT dimer (black). Substituted arginine R64 and the perturbed residues around the site of mutation are labelled.

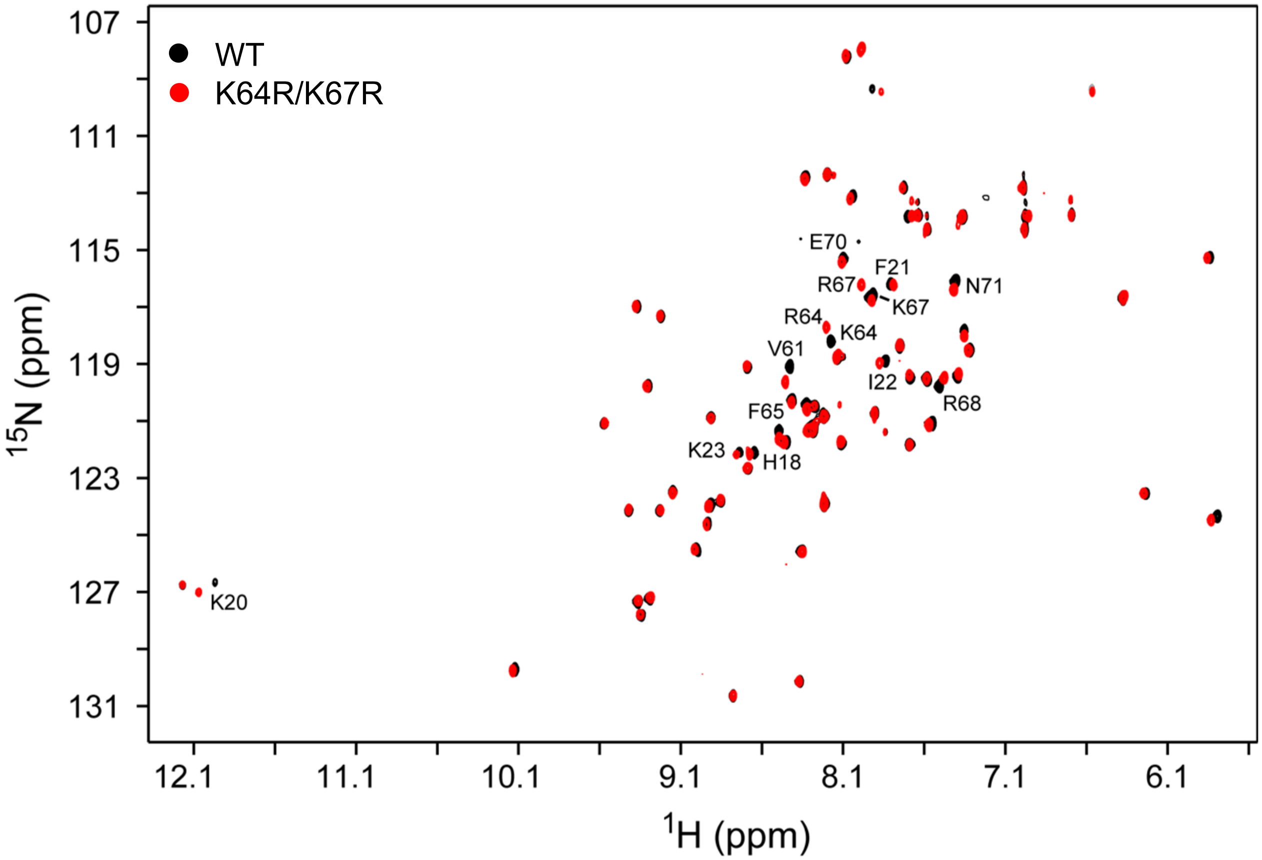

Figure S2. NMR structural characteristics of the K64R/K67R mutant. ^{1}H-^{15}N HSQC spectrum of K64R/K67R (red) overlaid on WT dimer (black). Substituted arginines R64, R67 and the perturbed residues around the site of mutation are labelled.

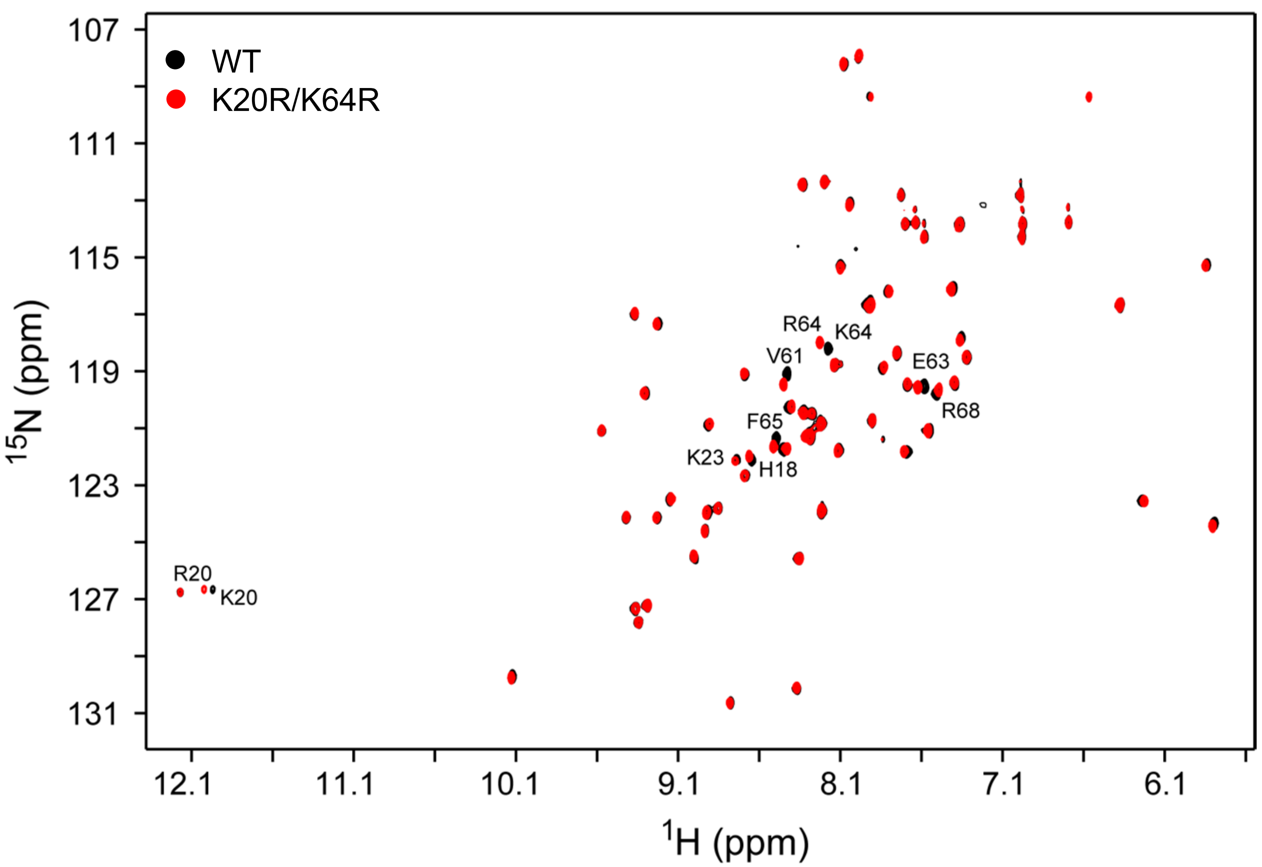

Figure S3. NMR structural characteristics of the K20R/K64R mutant. ^{1}H-^{15}N HSQC spectrum of K20R/K64R (red) overlaid on WT dimer (black). Substituted arginines R20, R64 and the perturbed residues around the site of mutation are labelled.

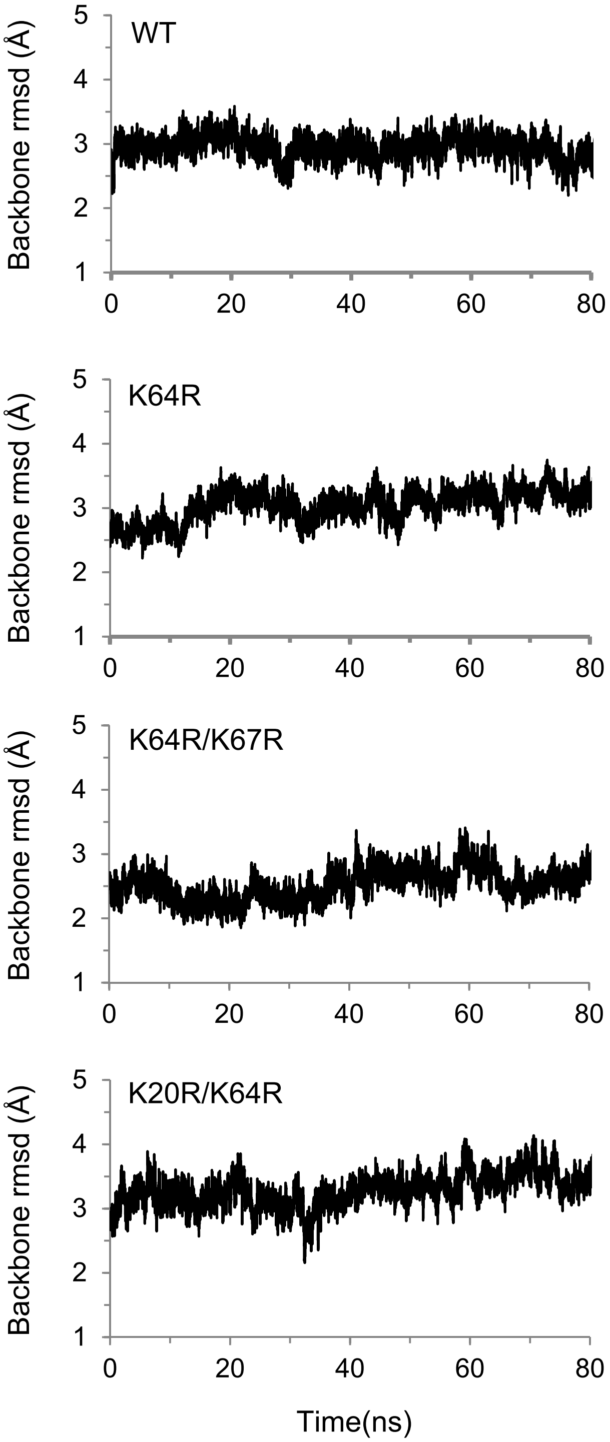

Figure S4. Backbone root mean square deviation (rmsd) of the MD trajectory in reference to the starting structures modelled from the NMR structure of CXCL8 dimer (PDB id : 1IL8). All the trajectories are stable and rmsd fluctuations are within 1-1.5 Å.
